# Dam and Dcm methylations prevent gene transfer into *Clostridium pasteurianum* NRRL B-598: development of methods for electrotransformation, conjugation, and sonoporation

**DOI:** 10.1186/s13068-016-0436-y

**Published:** 2016-01-20

**Authors:** Jan Kolek, Karel Sedlar, Ivo Provaznik, Petra Patakova

**Affiliations:** Department of Biotechnology, University of Chemistry and Technology Prague, Technicka 5, 166 28 Prague, Czech Republic; Department of Biomedical Engineering, Brno University of Technology, Technicka 12, 616 00 Brno, Czech Republic

**Keywords:** *Clostridium*, Butanol, Transformation, Conjugation, Methylation, Dam, Dcm, Electroporation, Sonoporation

## Abstract

**Background:**

Butanol is currently one of the most discussed biofuels. Its use provides many benefits in comparison to bio-ethanol, but the price of its fermentative production is still high. Genetic improvements could help solve many problems associated with butanol production during ABE fermentation, such as its toxicity, low concentration achievable in the cultivation medium, the need for a relatively expensive substrate, and many more. *Clostridium pasteurianum* NRRL B-598 is non-type strain producing butanol, acetone, and a negligible amount of ethanol. Its main benefits are high oxygen tolerance, utilization of a wide range of carbon and nitrogen sources, and the availability of its whole genome sequence. However, there is no established method for the transfer of foreign DNA into this strain; this is the next step necessary for progress in its use for butanol production.

**Results:**

We have described functional protocols for conjugation and transformation of the bio-butanol producer *C. pasteurianum* NRRL B-598 by foreign plasmid DNA. We show that the use of unmethylated plasmid DNA is necessary for efficient transformation or successful conjugation. Genes encoding DNA methylation and those for restriction-modification systems and antibiotic resistance were searched for in the whole genome sequence and their homologies with other clostridial bacteria were determined. Furthermore, activity of described novel type I restriction system was proved experimentally. The described electrotransformation protocol achieved an efficiency 1.2 × 10^2^ cfu/μg DNA after step-by-step optimization and an efficiency of 1.6 × 10^2^ cfu/μg DNA was achieved by the sonoporation technique using a standard laboratory ultrasound bath. The highest transformation efficiency was achieved using a combination of these approaches; sono/electroporation led to an increase in transformation efficiency, to 5.3 × 10^2^ cfu/μg DNA.

**Conclusions:**

Both Dam and Dcm methylations are detrimental for transformation of *C. pasteurianum* NRRL B-598. Methods for conjugation, electroporation, sonoporation, and a combined method for sono/electroporation were established for this strain. The methods described could be used for genetic improvement of this strain, which is suitable for bio-butanol production.

## Background

Interest in biofuel production, which could represent a useful substitute for standard fuels derived from fossil resources, has increased significantly over the last decade [1]. Butanol formed during acetone-butanol-ethanol (ABE) fermentation by solventogenic clostridia represents an interesting option for biofuel production, especially taking into account its physico–chemical properties that better suit requirements of gasoline motors compared to ethanol. Although butanol production by ABE has been known for more than 100 years [2], its industrial-scale production is hampered by a low final concentration, lower yield compared to ethanol, and in most species, an association of butanol production with sporulation. In addition, clostridia, including solventogenic species, are a polyphyletic group of bacteria, where transfer of knowledge gathered for one species, or even strain to another species/strain is difficult if not impossible. Most knowledge regarding the ABE process has been obtained from a single strain, *Clostridium acetobutylicum* ATCC 824, which differs in many features from other solventogenic clostridia [3]. Most other species, with the exception of *C. beijerinckii* NCIMB 8052 [4], have been described relatively poorly. These drawbacks have precluded the biotechnological production of bio-butanol on a larger scale [5]. Genetics and metabolic engineering represent new approaches with the possibility of significantly improving the ABE process.

The existence of methods for genetic manipulation of industrial microorganisms is generally essential for improving their properties to be appropriate for biofuel production. However, these methods are also very important for better, quicker and more effective research that could lead to the acquisition of important information useful in industrial processes. The most commonly used method for introducing foreign DNA into bacterial cells is transformation (an exogenous molecule of DNA is introduced directly through the cell membrane), conjugation (mediated by tight contact between donor-recipient cells and pili formation), and transduction (mediated by virus particles). In most cases, transformation of Gram-positive bacteria is more difficult compared to Gram-negatives and the development of transformation protocols is demanding. Gram-positive bacteria possess a thick peptidoglycan layer that is further enveloped by a protein S-layer and these bacteria also have only one cytoplasmic membrane, whose distortion can lead to immediate disruption of cell homeostasis and often death.

Transformation of gram-positive, strictly anaerobic bacteria of the genus *Clostridium*, is also usually accompanied by many drawbacks. For the introduction of foreign DNA into clostridial cells, several protocols have been described, based on conjugation with *Escherichia coli* [6, 7] or *Enterococcus* [8] donors, PEG-induced protoplast transformation [9, 10] and more recently, electroporation [11–14]. In addition, some less frequently used transformation approaches such as chemical treatment by Tris-PEG method [15] or sonoporation [16] have been tested.

Here, we describe the development of methods for genetic modification of *C. pasteurianum* NRRL B-598—a solventogenic bacterium producing butanol, acetone, and ethanol [17]. This strain is unique in its exceptional oxygen resistance, which is much higher than the standard butanol-producing model strains such as *C. pasteurianum* ATCC 6013, *C. beijerinckii* NCIMB 8052 or *C. acetobutylicum* ATCC 824. Also the whole genomic sequence is available for this strain [18, 19]. Moreover, only one system for genetic manipulation of *C. pasteurianum* species (type strain ATCC 6013) has been published [12]. We found that the development of methods for introducing DNA into the non-type, and at first sight untransformable, strain *C. pasteurianum* NRRL B-598, was problematic and completely different from other clostridia. We believe that our contribution to this field will strengthen knowledge on bacterial (especially *Clostridium*) transformation methods and encourage those who tackle similar tasks, trying to apply protocols developed for different species/strains, to their particular microorganisms.

## Results

### Initial transformation attempts

Initially, we conducted a series of pilot experiments based on previous descriptions of the transfer of foreign DNA to other clostridial species, as described in the literature [6, 8, 20, 21]. First, we tested various conditions for plasmid transfer by conjugation using various growth media (TYA, RCM, CBM, P2, YTG), time of conjugation (5–24 h), donor:recipient ratios (from 1:10 to 10:1) and, when no transformants resulted, electroporation was tested using various growth states of cells (OD 0.4–1.2), electroporation buffers (SMP, PEG, glycerol), cuvettes (0.2 and 0.4 cm gap), and electrical parameters (field strength 2.5–15 kV cm^−1^, time constant 5–20 ms). We also used plasmids from the pMTL80000 series encoding different replicons and antibiotic resistance markers [21]; this was to minimize the possibility that the plasmids may encode unsuitable origins of replication or antibiotic resistance for our strain. Unfortunately, no conditions that we tested during these pilot experiments led to successful transformation.

During pilot experiments, we discovered that strain *C. pasteurianum* NRRL B-598 was naturally resistant to chloramphenicol and thiamphenicol, therefore plasmids encoding thiamphenicol resistance, classically used as a selection marker for most clostridial strains, were not applicable. On the other hand, such a marker could be used for counter-selection during conjugation. We also verified that *C. pasteurianum* NRRL B-598 was not resistant to erythromycin or spectinomycin (20 μg/μl, 700 μg/μl resp.) at concentrations previously reported in the literature [21], but when a lower concentration of antibiotic was used, or too many cells were seeded onto agar plates, a very strong background growth was observed. Similarly, almost normal growth of cells was observed after longer periods (2–3 days) in TYA broth supplemented with appropriate concentrations of antibiotics.

### Bioinformatics analysis of the *C. pasteurianum* NRRL B-598 genome

Because all attempts at plasmid transformation of our strain failed, we decided to perform a more detailed bioinformatics analysis. The main purpose was to reveal genes encoding putative restriction-modification (R-M) systems that could present a problem during transformation of clostridia, and genes encoding putative DNA methyltransferases that could be connected with these R-M systems for protection of their own DNA [8, 12, 22, 23].

We took advantage of SMRT sequencing data used for the genome assembly [19] to study DNA methylation on a genome-wide scale. We analyzed all base modifications to determine modified sequence motifs. Out of the total, 2033 positions in the *C. pasteurianum* NRRL B-598 genome were detected as being methylated (m4C or m6A) with the majority being m6A methylations (1996 positions). Both detected motifs (GA**A**YNNNNNNNR**T**ANYC, G**A**YNNNNNNC**T**AG) demonstrated novel recognition sequences that have not been described previously. Letters in bold denote methylated bases. Highlighted ‘T’ represents methylation of ‘A’ in the opposite strand.

The data were deposited in the REBASE PacBio database (http://rebase.neb.com/cgi-bin/pacbiolist) [24] and were connected to the R-M system based on homology searching. The detected methylation motifs, both m6A types, are summarized in Table 1, along with the corresponding methyl transferase (MT)-encoding genes.

**Table 1:** Methylated motif detected for *C. pasteurianum* NRRL B-598

R-M system type	Motifs (±strand)	No. in genome	No. detected (±strand)	% detected (±strand)	Locus tag	Nomenclature
I	GRNTAYNNNNNNNRTTC/GAAYNNNNNNNRTANYC	406	385/380	94.83/93.60	X276_10630	M.Cpa598I
I	CTAGNNNNNNRTC/GAYNNNNNNCTAG	606	573/560	94.55/92.41	X276_12360	M.Cpa598II

In addition to above-mentioned type I R-M systems, three more putative R-M systems were predicted, including two type II R-M systems and a single type IV R-M system. A summary of all five systems is found in Table 2. BLAST results also showed that no genes homologous to *E. coli* Dam and Dcm were present in the *C. pasteurianum* NRRL B-598 genome.

**Table 2:** R-M systems in *C. pasteurianum* NRRL B-598 genome

Type	Name	Gene^a^	Meth. type	Recognition	Locus (X276_)	Most similar (% identity)
I	Cpa598IP	R	m6A	GAAYNNNNNNNRTANYC	10620	CspMORF4102P (95 %)
	M.Cpa598I	M			10630	M.CbeG117ORFCP (97 %)
	S.Cpa598I	S			10635	S.CspMORF4102P (49 %)
I	Cpa598IIP	R	m6A	GAYNNNNNNCTAG	12355	Csc25775ORFJP (90 %)
	M.Cpa598II	M			12360	M.Csc25775ORFJP (96 %)
	S.Cpa598II	S			12365	S.Bme201ORFGP (56 %)
II	M1.Cpa598ORF20205	M	m5C	–	–	M1.CboKAPB3ORF12160P (87 %)
	M2.Cpa598ORF20205	M			01545	M2.CboKAPB3ORF12160P (84 %)
	R1.Cpa598ORF20205	R			–	R2.Cce743ORF4007P (46 %)
	R2.Cpa598ORF20205	R			01555	R1.Bce3081ORF2217P (31 %)
II	M.Cpa598ORF2410P	M	m6A	GATC^b^	20735	M.Cbe59BORF1284P (100 %)
IV	Cpa598ORF12465P	R	–	–	12465	Cdi15410ORFAP (93 %)

^a^ *R* restriction endonuclease, *M* restriction endonuclease coupled methylation protein, *S* R-M specific protein

^b^ Predicted recognition site

**Fig. 1:**
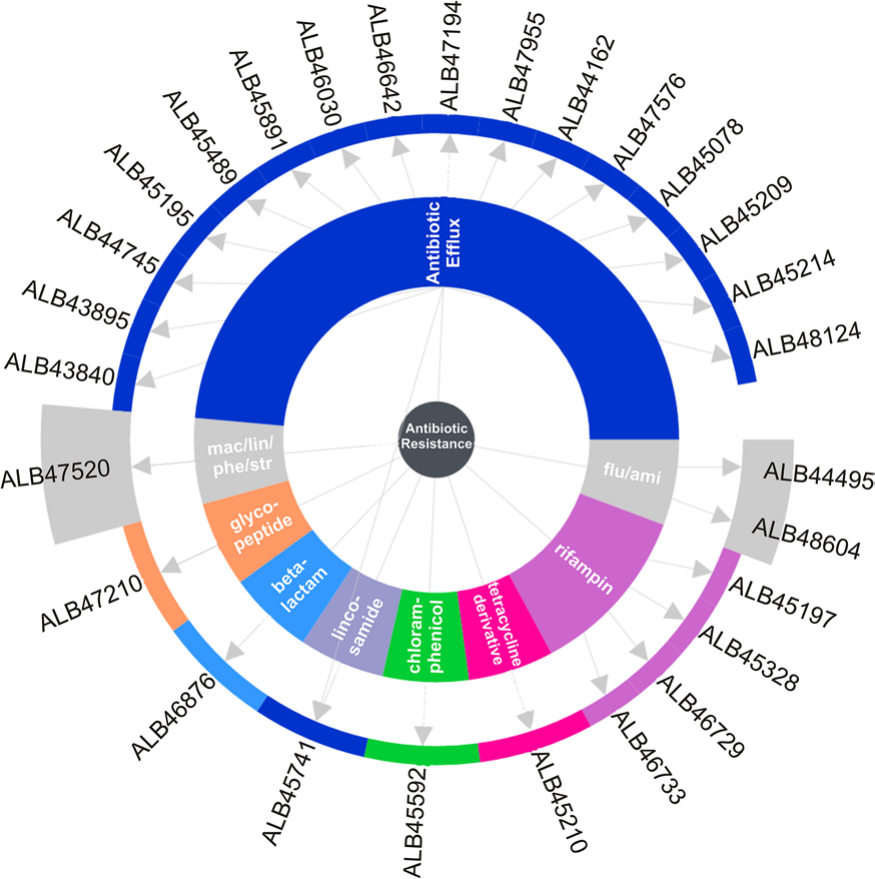
Antibiotic resistance genes in *C. pasteurianum* NRRL B-598 genome. Overall resistance in the *center*, resistance classes in the *middle*, and individual resistance genes (and their NCBI accession numbers) on the *outer ring*

We also searched for antibiotic resistance genes. In total, 28 ORFs with antibiotic resistance functions, divided into nine resistance classes, were identified in the genome. All of these ORFs were assigned GenBank accession numbers for the relevant protein product (Fig. 1). As expected, we verified the presence of a gene for chloramphenicol acetyltransferase (cat, [GenBank: ALB45592]) that encoded resistance to chloramphenicol and thiamphenicol, as observed during our experiments. Moreover, genes encoding erythromycin or spectinomycin resistance were not identified. A substantial part of the antibiotic resistance of *C. pasteurianum* NRRL B-598 is mediated by an antibiotic efflux system.

### Investigation of potential restriction barriers

As described previously, nucleases can be located on the surface of cells and in some cases and degradation of DNA can already start after adding DNA to the cells [25]. In other cases, enzymes with nuclease activity are located in the cytoplasm. Hence we examined nuclease activities in both the protoplast crude lysate (without any parts of the cell envelope) as well as in the whole cell extract.

**Fig. 2:**
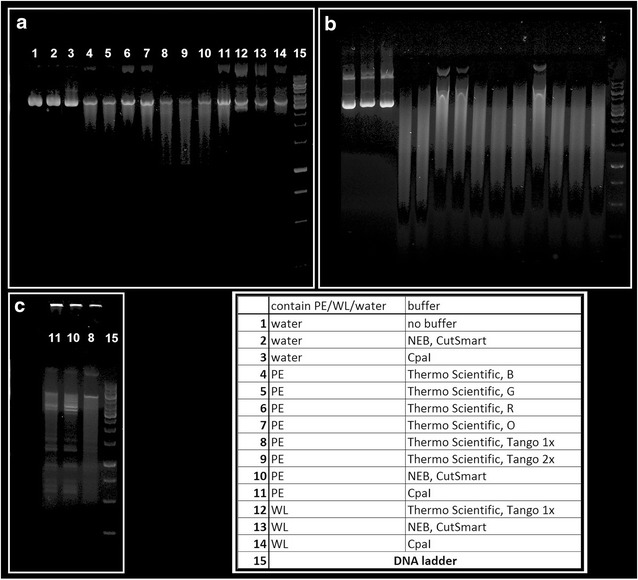
Testing the presence of potential restriction barriers. Cultivation of pMTL83253 (**a**) and pMTL82254 (**b**) with crude protoplast extract (*PE*) and whole cell lysate (*WL*) prepared from *C. pasteurianum* NRRL B-598 at 37 °C. Positive control (**c**): cultivation of pMTL83253 with *PE* prepared from *C. pasteurianum* DSM 525 by the same method

We did not detect any restriction activity when pMTL83253 (plasmid does not contain motifs of predicted type I R-M systems) was incubated with crude extracts and whole cell lysate. In the case of pMTL82254 (contains one of each predicted motifs), plasmid DNA was nearly completely digested in broad spectrum of cultivation conditions. Restriction did not provide separate bands (DNA fragments) like in case of cultivation with crude extract from *C. pasteurianum* DSM 525, but led to one fuzzy smear (see Fig. 2). The same restriction pattern was obtained at 30 and 37 °C.

### Influence of methylation and establishment of an electroporation protocol

**Fig. 3:**
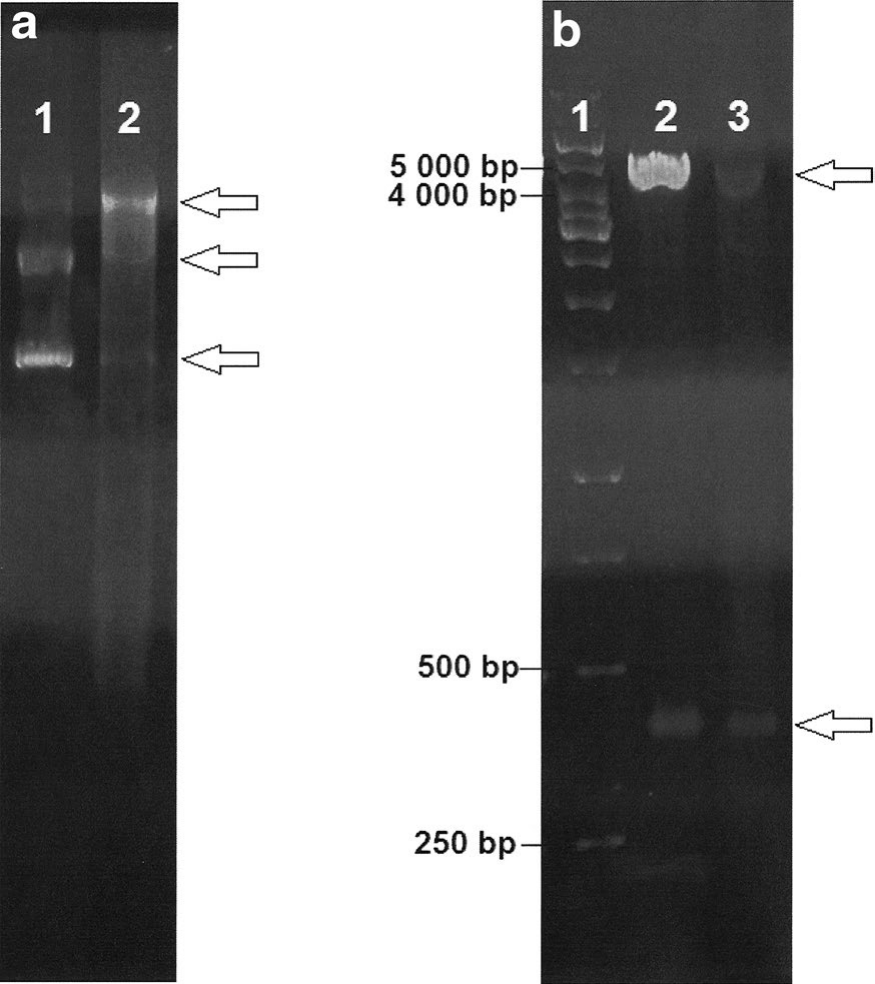
Confirmation of pMTL83253 presence in *C. pasteurianum* NRRL B-598 erythromycin-resistant transformants. **a** pMTL83253 isolated from *E. coli* JM110 (*a1*) and *C. pasteurianum* NRRL B-598 transformants (*a2*). **b** pMTL83253 isolated from *E. coli* JM110 (*b2*) and *C. pasteurianum* NRRL B-598 transformants (*b3*) cleaved by *Pst*I (resulting fragments 370 bp and 4413 bp) compared to the GeneRuler 1 kb DNA ladder - Thermo Scientific (*b1*)

As a next step, we wanted to test whether plasmid DNA without Dam and Dcm methylation could be used for transformation. We extracted plasmids from *E. coli* JM110 (*dam*−/*dcm*−), a strain used for preparation of unmethylated DNA. After pilot electrotransformation experiments using unmethylated pMTL83253 (containing the pCB102 origin derived from *C. butyricum*) and conditions described previously for *C. beijerinckii* [25], a few erythromycin-resistant colonies (1–12 CFU) were obtained after 48 h of growth on selective agar medium. Also other tested plasmids (pMTL83353-pCB102 replicon and spectinomycin selection marker; pMTL82251-pBP1 replicon; pMTL84251-pCD6 replicon; pMTL85251-pIM13 replicon) were transformed successfully but the CFU yields were much lower (a maximum of 4 CFU). Because of the best transformation efficiency achieved, as well as the fact that the pCB102 origin is the replicon that is used, for example, in standard pMTL007 plasmids (ClosTron system) [7] used for fast and specific knock-outs, we performed all following experiments with pMTL83253. The presence of pMTL83253 in erythromycin-resistant colonies was verified by its isolation and restriction digestion by *Pst*I. Bands of the digested DNA were compared to bands of pMTL83253 isolated from *E. coli* and digested in the same way (Fig. 3). The presence of pMTL83253 was confirmed in all erythromycin-resistant colonies that we tested.

After achieving successful transformation, we aimed to improve transformation efficiency for unmethylated plasmid DNA because the twelve colonies observed (observed maximum) corresponded to a transformation efficiency of only 6 cfu/μg DNA, which is very low and would not be compatible with the use of this method for genetic manipulations.

**Fig. 4:**
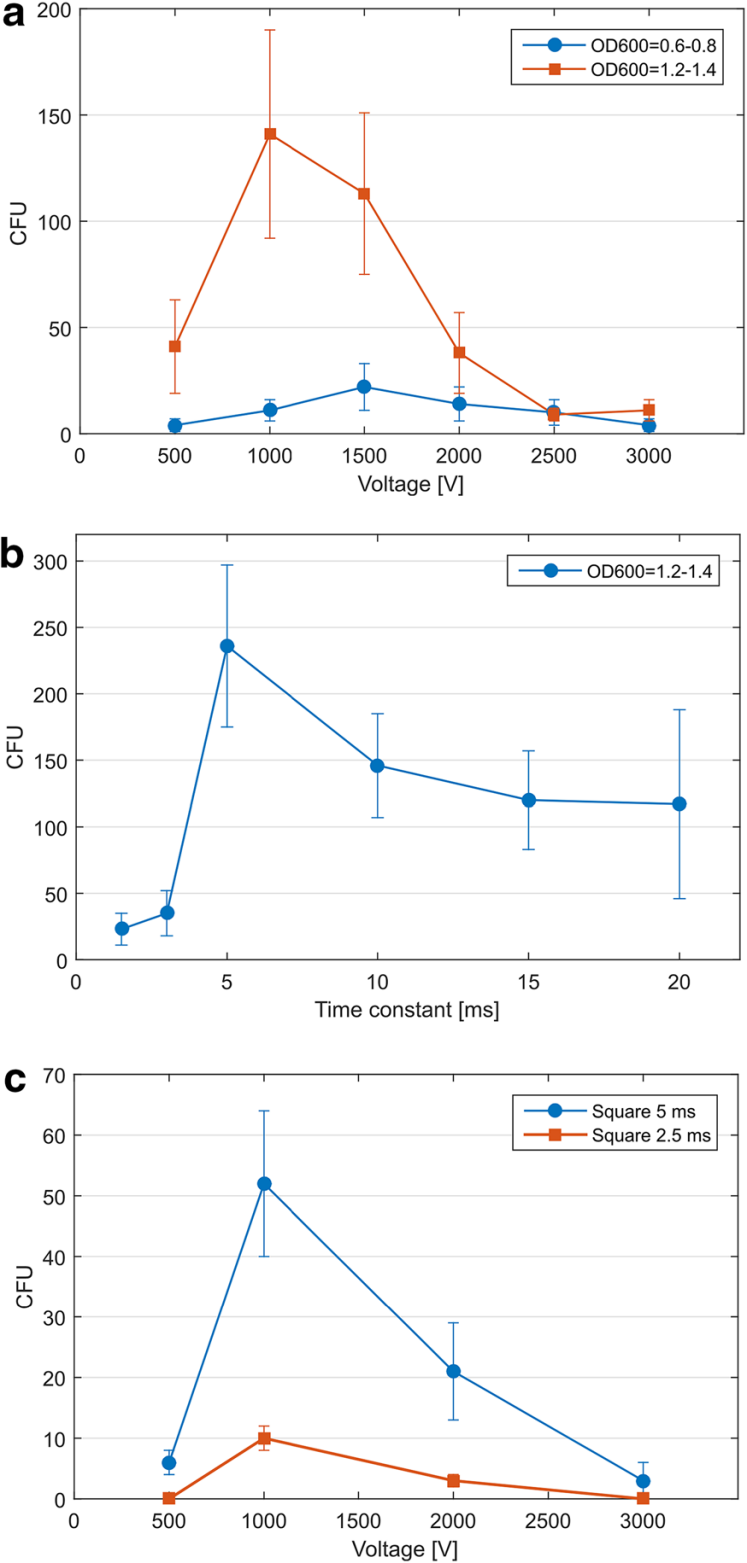
Optimization of electrotransformation conditions. Influence of various voltages and cell growth state presented by OD_600_ (used conditions: 0.2 cm gap electroporation cuvette, time constant 11 ms) on transformation efficiency (**a**); influence of different time constants (conditions used: 0.2 cm gap electroporation cuvette, voltage 1 000 V) on transformation efficiency (**b**); influence of square-wave pulse delivery (**c**)

Initially, we tested different voltages (2500–15,000 V cm^−1^). A second parameter, investigated and optimized during the first experiments, was the growth state of the cells, represented by culture optical density. For this purpose, we prepared electrocompetent cells from cultures of different OD_600_ (0.6–0.8 and 1.2–1.4), representing the previously used states of culture for electrotransformation of clostridia. When cells at an OD_600_ of around 1.2–1.4 were used, transformation efficiency was significantly improved (Fig. 4). In the following electroporation experiments, time constant, as the main parameter of electroporation, was investigated using the best voltage and cell growth conditions (see above). We observed that shorter electric pulses (5 ms) were significantly better for transformation efficiency compared to higher values. CFUs obtained using different time constants are shown in Fig. 4. Square-wave pulse delivery was also tested, but transformation efficiencies were significantly lower than with exponential pulse mode (see Fig. 4).

We also tested a set of various electroporation buffers (30 % PEG 8000 and SMP buffer at different pH values). However, no increase in transformation efficiency was obtained in any other buffers during these experiments. The addition of cell-wall weakening additives (different concentrations of glycine, ampicillin or Tween 80) or treatments with various concentrations of lysozyme prior to electroporation, which have been described previously [12, 26] as methods for significantly increasing transformation efficiency in Gram-positive bacteria, was not successful and no transformants or poor transformation efficiencies were observed (data not shown). Generally, very poor growth was observed in the presence of low concentrations of glycine (more than 0.25 %), even with sucrose or PEG osmotic protection. Equally, addition of osmoprotective agents (various concentrations of sucrose, PEG or lactose) to the recovery medium always had detrimental effects on growth and transformation efficiency, and addition of sucrose to the growth medium at high concentrations (0.2 M and more) led to a significant decrease in growth. Importantly, when culture degeneration [27] was observed (represented mainly by formation of very long, mycelium-like cells in log and late-log phase), transformation efficiency was reduced drastically and only a few colonies grew on the selective medium.

After optimization of electrotransformation steps, we wanted to better understand the influence of Dam and Dcm methylation individually to resolve which one is detrimental or potentially helpful in transformation. We compared electroporation transformation efficiencies of experiments where plasmid DNA isolated from the following methylation-deficient *E. coli* strains were used: JM110 (*dam*−/*dcm−*), BL21 (*dam*+/*dcm*−) and GM33 (*dam*−/*dcm*+). DNA extracted from *E. coli* DH5α (*dam*+/*dcm*+) was also used for confirmation that Dam and Dcm methylations represent a real obstacle to transformation, even when the optimized electrotransformation protocol was performed. A few erythromycin-resistant colonies (a maximum 8 of CFU) containing pMTL83253 were sometimes obtained if DNA from DH5α (fully methylated) was transformed. Relatively consistent results were achieved by transformation of hemimethylated plasmid DNA. Both methylations led to a significant reduction in transformation efficiency. The influence of various methylations on electrotransformation efficiencies is summarized in Table 3.

**Table 3:** Influence of DNA methylation stage to the electrotransformation efficiency

DNA amount [μg]/*E. coli* strain (designation)	CFU (average count)^a^	Efficiency (CFU per μg DNA)
2 μg/DH5α (*dam* +/*dcm* +)	3	1.5
2 μg/GM33 (*dam* −/*dcm* +)	27	13.5
2 μg/BL21 (*dam* +/*dcm* −)	28	14
2 μg/JM110 (*dam* −/*dcm* −)	236	118

^a^2 μg of DNA was used for transformation

### Establishment of conjugational transfer

Conjugation was not observed when an *E. coli* strain supporting Dam or Dcm methylation was used as a donor for transmission of pMTL80000 series plasmids to our strain used in the pilot experiment (see above). Based on our experience from electrotransformation experiments, we constructed a new conjugation donor strain by transmission of RP4 helper plasmid to *E. coli* JM110 (*dam*−/*dcm*−) containing pMTL83253. With this donor ensuring transfer of unmethylated pMTL83253 between donor and recipient cells, we tested for conjugation. Conjugation using a methylation-deficient donor was successful and many erythromycin-resistant colonies were observed after 48 h. CFUs achieved after various conjugation times (6 or 24 h) are summarized in Table 4.

**Table 4:** Summary of pMTL83353 containing CFU yielded by conjugation, sonoporation, and combined sono/electroporation approaches

Method	CFU (average count)	Efficiency (CFU per μg DNA)
Conjugation (*E. coli* JM110 containing RP4 and pMTL83253 donor)		
6 h of conjugation	12	
24 h of conjugation	37	
Sonoporation		
10 % PEG 8000 buffer, 20 s pulse	225^a^	112.5
30 % PEG 8000 buffer, 20 s pulse	321^a^	160.5
Sono/electroporation		
30 % PEG 8000 buffer, 20 s ultrasound pulse, 5 ms square-wave pulse 5 ms (1250 V)	265^b^	530

^a^2 μg of DNA was used for transformation

^b^0.5 μg of DNA was used for transformation

### Use of sonoporation for transmission of plasmid DNA

As described previously, ultrasound could also be a useful technique to use for transformation of Gram-positive bacteria. From a few sonoporation media tested (TYA broth, 0.5 M CaCl_2_, sterile water, SMP and PEG), only 10 and 30 % PEG 8000 were suitable for relatively high-efficiency transformation. No or only a few transformants were achieved when other sonoporation media were used. An adequate time of ultrasonic pulse was designed according to previous experiences with sonoporation of Gram-positive bacteria, where 20 s was identified as a critical time for ultrasound-mediated plasmid DNA degradation but less time led to a reduction in transformation efficiency [16]. Sonoporation has been proven to be a very effective method of transformation that provides even higher transformation efficiencies than electrotransformation. Efficiencies of transformation achieved by sonoporation are summarized in Table 4.

### Combined sono/electroporation for increased transformation efficiency

**Fig. 5:**
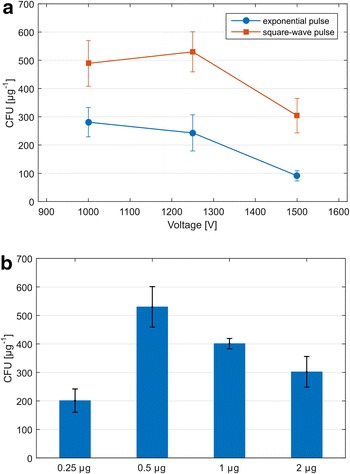
Optimization of sono/electroporation conditions. Influence of various voltages and exponential and square-wave pulse deliveries (**a**); influence of DNA amount on transformation efficiency (**b**)

Because cell-wall weakening approaches were not successful, we compiled a combined method using both sono- and electroporation for improving transformation efficiencies. During the first set of sono/electroporation experiments, we observed that a square-wave pulse provided more consistent results and significantly higher efficiency than the previously used exponential pulse. Also, different amounts of DNA (0.25–2 μg) were used for establishing the most efficient approach. Slightly higher voltage (1250 V) produced the most transformants in the square-wave mode and best transformation efficiency was achieved with 0.5 μg of plasmid DNA (see Fig. 5). By a combination of both techniques, we were able to reach a transformation efficiency of 5.3 × 10^2^ cfu/μg DNA (see Table 4).

## Discussion

The development of methods for efficient genetic manipulation of clostridial bacteria is generally very challenging. Protocols for transmission of foreign DNA to many clostridial species have been developed [20], but these transformation procedures use very different conditions and their overall efficiencies vary by orders of magnitude from 10^0^ to 10^6^ transformants/μg of DNA. Furthermore, transformation conditions are often useful for only one strain and cannot readily be used for other species or even strains. At least, rational step-by-step optimization of the protocol is necessary in order to achieve consistent results. A unique approach to transformation must be developed when the strain expresses a specific restriction barrier that prevents effective transformation, or when conditions from previously published approaches are unsuccessful, as in our case.

*C. pasteurianum* NRRL B-598 represents a non-type strain of solventogenic clostridium that could be a good candidate for production of organic solvents in an ABE process. This strain excels in very high oxygen resistance and overall robustness that could be helpful for a large-scale ABE process. Moreover, biosynthesis of some nonspecific proteases that allows the use of cheap nitrogen sources in its cultivation (e.g., waste whey products) has been described previously for this strain [28]. During our experiments, we showed that *C. pasteurianum* NRRL B-598 carries a *cat* gene encoding resistance to chloramphenicol and thiamphenicol, the antibiotics that is normally effective against many strains of clostridium bacteria. This finding is a little surprising because chloramphenicol and thiamphenicol resistances have only been observed in solventogenic species such as *C. beijerinckii*, but not *C. pasteurianum*.

The action of various restriction-modification (R-M) systems represents a frequent obstacle in the transformation of clostridia, as well as other Gram-positive species. Type II R-M systems recognize a defined short sequence in the foreign DNA and promote its degradation after transmission to the cytoplasm, or even immediately on the cell surface [29]. R-M II systems were described as a reason preventing transformation of *C. acetobutylicum* ATCC 824 [22], *C. pasteurianum* ATCC 6013 [12] or *C. cellulolyticum* ATCC 35319 [8]. In these cases, special treatment by DNA-methyltransferase, which masks all recognition sequences, was necessary before transformation. Type I R-M systems could also be responsible for a decrease in transformation efficiency like in *C. saccharobutylicum* NCP 262 [23]. Specific protein inhibitors (such as TypeOne restriction inhibitor), protective methylation or heat inactivation could be approaches for overcoming these systems [29]. Equally, reduction of transformation efficiency could be caused by R-M III or IV, but these systems have, so far, been very poorly described in clostridia.

Based on the analysis of PacBio SMRT data, we demonstrated the genomic existence of two type I R-M systems, Cpa598I and Cpa598II. Activity of these systems was also confirmed experimentally by cultivation of pMTL82254 which contained recognition sequences of both R-M systems. Restriction provides probably unspecific cleaving of DNA in the direction from the recognized motifs which is typical for type I R-M systems [30]. Both recognized motifs are included in sequence of pBP1 replication origin module of pMTL80000 plasmids system thus it is better to use other replicon for transformation of this strain. On the other hand, when unmethylated pBP1 replicon-based plasmid (pMTL82251) was transformed by electroporation, we were still able to obtain a few transformants.

Both type II R-M systems are most certainly inactive because no methylated recognition sequence for Cpa598ORF2410 system was found and no m5C methylations assigned to Cpa598ORF20205 system were detected. We note that the kinetic signatures of m5C bases may not have been strong enough to study properly, but in a relatively high sequence coverage (79×) not a single m5C methylation was detected and also no active type II R-M system was obtained during experimental testing of their presence in the protoplast or whole cell lysates. Activity of the remaining type IV R-M system remains unclear, since these systems are poorly described and neither recognition sequence nor the type of methylation was assigned to this system. Nevertheless, because Cpa598ORF12465P is a methyl-directed restriction enzyme, its activity could also be the reason for decreased transformation efficiency. Further studies are required to verify these hypotheses.

The *C. pasteurianum* NRRL B-598 genome contains a relatively large number of antibiotic efflux genes. Antibiotic resistance can be confirmed by almost normal growth of cells in a medium containing various antibiotics over long periods of time.

The addition of TypeOne restriction inhibitor, which has been described previously as a functional agent for overcoming R-M I systems in *E. coli* or *Salmonella typhimurium* [31], also did not lead to successful transformation. Based on these results, we assumed that a restriction barrier requiring methylation protection of plasmid DNA probably did not constitute a relevant obstacle during transformation of DNA extracted from *E. coli* or its conjugal transfer to *C. pasteurianum* NRRL B-598.

Methylation of transmitted DNA can also clearly affect the efficiency of bacterial transformation. Significant reductions in transformation efficiencies when methylated DNA was used were described for many bacterial species such as *Streptomyces* or *Lactobacillus*. Methyl-specific restriction systems probably play a major role in these observations [32, 33], but the fact that methylated *ori* sequences on a plasmid may not associate with a specific replication protein could also play an important role in transformation efficiency [34]. Fully methylated DNA isolated from *Escherichia coli* (*dam*+/*dcm*+) was, in most cases, referred to as the best template for clostridial transformation because Dam and Dcm methylation could protect DNA from degradation by nucleases and could increase clostridial transformation efficiencies. Reported cases of detrimental influences of *E. coli* methylation were observed in *C. thermocellum* DSM1313 and *C. ljungdahlii* DSM 13528, but eventually only Dcm methylation was identified as the origin of transformation problems in both experiments [13, 34]. Surprisingly, when unmethylated plasmid DNA was used for electrotransformation of *C. pasteurianum* NRRL B-598, we suddenly obtained a few transformants. For electrotransformation, a previously published protocol for *C. beijerinckii* NCIMB 8052 [25] was used and the maximum transformation efficiency, achieved with pMTL82353, was 6 cfu/μg DNA. The transformation efficiency achieved was very low compared to other clostridia or Gram-positive bacteria and could not be used for effective genetic manipulations or research on this strain. Because a previously published protocol for other species was used without changes, we wanted to optimize it directly for *C. pasteurianum* NRRL B-598, hopefully leading to an improved transformation efficiency.

The efficiency of electrotransformation may be affected by many parameters such as growth medium, cell growth phase, composition of electroporation buffer, voltage of electric pulse, or its length (influenced mainly by capacitance and resistance of the electroporator). For electrotransformation of clostridial species, cells in early-log to late-log growth phase, different electroporation buffers with low conductivity containing osmostabilizing agents (sucrose, PEG, etc.), and a relatively low electric field (around 5 kV cm^−1^) are usually used [20]. We found that the best growth phase of *C. pasteurianum* NRRL B-598 for electrotransformation was between late logarithmic and early-stationary phase (OD_600_ 1.2–1.4), which is not typical for most solventogenic strains. Similarly, the best transformation efficiency was obtained when electroporation was conducted in 10 % PEG 8000 and decreased when the SMP electroporation buffer (at various pH values) was used. Through step-by-step optimization, we were able to achieve an average electrotransformation efficiency of 1.2 × 10^2^ cfu/μg DNA when unmethylated DNA was used. This was much lower than for the type strains *C. acetobutylicum* or *C. beijerinckii,* where the electrotransformation efficiencies reached 10^4^–10^5^ transformants per μg of DNA [22, 25]. Nevertheless, this efficiency is sufficient to use this method for some genetic improvements and basic research on this intractable strain.

Achieved transformation efficiency showed clearly that with a decreasing number of any *E. coli* methylations, transformation efficiency significantly increased. Thus, both Dam and Dcm methylations were shown to be detrimental to transformation, a fact that has not been described previously in transformation of other clostridia. Previously, Pyne et al. [20] described similar effect of CpG methylation which presence led to obtain no transformants even though CpG provided good protection against digestion by described R-M system. If we take into account the number of Dam- and Dcm-specific methylation sites on pMTL83253 (10 and 18 resp.), we can postulate that Dam methylation could be a little more detrimental than Dcm, which is at variance with findings obtained previously [13, 35]. Decreased efficiency could be caused by a reduction in replication efficiency or some methyl-specific restriction system that may be present in cells as a protection against foreign DNA, e.g., bacteriophage, exhibiting a foreign methylation pattern. The best described similar systems are, for example, the *Dpn*I system in *Streptococcus pneumoniae* [36] or model methylation-dependent systems *Mcr*A, *Mcr*BC, and *Mrr* as described in *E. coli* [30]. If some methyl-specific type IV restriction system occurs in our strain (see above), it would be quite interesting because no restrictions were obtained when we conducted an examination of restriction systems with Dam and Dcm methylated pMTL83253. However, we focused mainly on R-M I and II systems, so some putative R-M IV (methyl-specific) systems may not be active under these in vitro conditions.

The influence of *E. coli* methylation was also verified in conjugation experiments, where pMTL82353 transmission was only successful in the methylation-deficient donor strain (JM110 containing RP4). The existence of effective conjugal transfer could be very useful because it represents an effective way to transfer large plasmids to *C. pasteurianum* NRRL B-598, which is poorly transformable by electroporation and sonoporation techniques. No evidence concerning the use of a conjugation donor mediating transfer of unmethylated DNA between *E. coli* and clostridia has been published previously and this method could represent a fast and relatively easy method for an initial examination of the influence of methylation on transmission efficiency because this IncP-based conjugation method is applicable for many clostridial species in a similar arrangements of experiments.

Sonoporation is a relatively new method that is not used frequently for bacterial transformation. It is probably based on the cavitation of the cell wall and membrane, mediated by ultrasound pulse delivery that results in transmission of DNA into the cell [37]. Historically, a few transformations of thermophilic clostridia were conducted successfully using ultrasound-mediated transfer [16]. We were able to transform *C. pasteurianum* NRRL B-598 by sonoporation using a simple 20-s ultrasound pulse. Surprisingly, the average efficiency of pMTL82353 transfer was 1.6 × 10^2^ cfu/μg DNA, which was even more efficient than electrotransformation. Moreover, sonoporation is a method that does not require any special or expensive equipment and is fast and reliable. On the other hand, it is likely that ultrasound-mediated transformation is limited by the size of the transferred plasmid because larger plasmids can be more rapidly destroyed by sonication. Polyethylene glycol probably plays an important role in transformation of *C. pasteurianum* NRRL B-598 because it may act as an osmostabilizer and also as an agent ensuring easier transmission through the bacterial membrane. Sonoporation of unmethylated DNA was the necessary condition and when DNA extracted from DH5α was used, no or only a few transformants were obtained.

Ultrasound pre-treatment prior to electrotransformation was used previously e.g., for *Saccharopolyspora erythraea* [38] or *Streptomyces spp.* [39]. Ultrasound can effectively disorganize the cell wall; therefore, it may be useful to increase the efficiency of transformation. Because we were not successful using standard cell-wall weakening procedures (glycine addition or lysozyme treatment), we attempted to enhance the uptake of DNA into bacterial cells by sonication prior to electroporation, especially in this case where sonication was proven to be the best approach for transformation. Sono/electroporation proved to be the best method for transformation of *C. pasteurianum* NRRL B-598, producing relatively consistent results over many replicates. It was shown to be important to use a square-wave pulse during sono/electroporation because when a standard exponential pulse was delivered, transformation efficiency decreased. This was mainly result of higher cell mortality probably due to ultrasound-caused cell wall disturbances. By the combination of both methods, we were able to achieve transformation efficiency of 5.3 × 10^2^ cfu/μg DNA, which was about four times higher than using sonoporation or electroporation alone.

The transformation efficiency that was achieved is sufficient for effective plasmid DNA delivery to *C. pasteurianum* NRRL B-598 and could be used, for example, for simple gene over-expression or knock-out experiments. Due to restrictions with equipment, all transformation steps (electroporation, sonoporation, and partial culture manipulation) were performed outside of the anaerobic chamber. We assume that strict anaerobic conditions may improve the efficiency of DNA transmission however even under the described conditions, we were able to achieve usable and repeatable results for this oxygen resistant strain. It is also possible that less well-described *E. coli* methylases (e.g., genomic orphan methylases) could be responsible for the relatively low efficiency of DNA transmission and could be the subject of further research.

## Conclusions

We have described methods for transmission of foreign DNA to *C. pasteurianum* NRRL B-598 for future potential genetic manipulation. Using PacBio kinetic data, we described 2 previously unknown recognition motifs for type I R-M systems in the *C. pasteurianum* NRRL B-598 genome as well as demonstrated the inactivity of 2 type II R-M systems. We also discovered a putative type IV methyl-directed R-M system that could be responsible for low transformation efficiency. Transformation or conjugal transfer of non-methylated DNA was necessary for high-efficiency transmission by all methods tested, which is unusual for clostridial transformation methods described to date. Methods for conjugation, electrotransformation, not frequently used sonoporation, and even their combination (sono/electroporation) were described and a maximum transformation efficiency of 5.3 × 10^2^ cfu/μg DNA was achieved. In this paper, we also demonstrated that development of genetic methods for a non-type strain could be challenging and be completely different to the type strain or even other clostridia. All described methods could lead to more effective research that would make this strain useful in biofuel production. This work also reveals new knowledge about the diversity of defense mechanisms against foreign DNA in solventogenic clostridia and shows the possibility of using sono/electroporation for efficient transformation of Gram-positive bacteria.

## Methods

### Bacterial strains and growth conditions

All strains described in this paper are summarized in Table 5. *C. pasteurianum* NRRL B-598 was maintained as a spore suspension in sterile distilled water and grown in TYA medium [40] containing in g/l: 20 glucose; 2 yeast extract (Merck); 6 tryptone (Sigma); 0.5 KH_2_PO_4_; 3 ammonium acetate; 0.3 MgSO_4_.7H_2_O; 0.01 FeSO_4_. TYA plates (solidified by 1.5 % agar) were supplemented with erythromycin (20 μg/ml), spectinomycin (700 μg/ml), chloramphenicol (25 μg/ml), or thiamphenicol (15 μg/ml) as required. *C. pasteurianum* DSM 525 was cryopreserved in 30 % glycerol solution (maintained in −80 °C) and grown in RCM broth (Merck) supplemented by glucose to a final concentration of 20 g/l. Cultivation of both strains was performed in an anaerobic chamber (Concept 400; Ruskinn Technology, UK) in a stable atmosphere of 95 % N_2_/5 % H_2_ and at 37 °C. Clostridium basal medium (CBM) [41], semi-defined P2 medium [42], and YTG [43] media were also used during this study.

**Table 5:** Summary of bacterial strains and plasmid DNA used in this thesis

Bacterial strains	Genotype	Source
*Clostridium pasteurianum* NRRL B-598		ARL collection (NRRL)
* Clostridium pasteurianum* DSM 525 (ATCC 6013)	Coding type II restriction system CpaAI	DSMZ
* Escherichia coli* DH5α (DSM 6897)	*dam* +/*dcm*+	DSMZ
* Escherichia coli* JM110 (DSM 11539)	*dam*−/*dcm*−	DSMZ
* Escherichia coli* BL21(DE3)	*dam*+/*dcm*−	CGSC
* Escherichia coli* GM33	*dam*−/*dcm*+	CGSC
* Escherichia coli* HB101 (DSM 1607)	*dam*+/*dcm*+	DSMZ
*E. coli* plasmids		
RP4 (RK2)	Coding IncP-based conjugation function	DSMZ
*E. coli*/*Clostridium* shuttle plasmids		
pMTL83353	*aad9*, pCB102 origin of replication	[21]
pMTL82251	*ermB*, pBP1 origin of replication	[21]
pMTL83253	*ermB*, pCB102 origin of replication	[21]
pMTL84251	*ermB*, pCD6 origin of replication	[21]
pMTL85251	*ermB*, pIM13 origin of replication	[21]

All *E. coli* strains were cryopreserved in 20 % glycerol solution (maintained in −80 °C) and grown on LB medium (containing in g/l: 10 tryptone; 5 yeast extract; 5 NaCl) in 37 °C. LB broth or plates (1.5 % agar) were supplemented with erythromycin (500 μg/ml), spectinomycin (100 μg/ml), ampicillin (100 μg/ml), or streptomycin (30 μg/ml) as necessary.

### Plasmids, oligonucleotides, and DNA manipulation

All plasmids used in this paper are summarized in Table 5. Plasmid DNA was transmitted to *E. coli* strains by standard CaCl_2_ treatment; transmission of RP4 helper plasmid between *E. coli* strains was performed by conjugation. For isolation of plasmid DNA, a High Pure Plasmid Isolation Kit miniprep (Roche, Switzerland) was used. Plasmid DNA from *C. pasteurianum* NRRL B-598 was extracted by the method described previously for *C. pasteurianum* ATCC 6013 [12] with modifications. For isolation, 8 ml of culture (OD_600_ ca. 1.3–1.5) was harvested by centrifugation (10,000×*g*, 2 min.), washed once in 1.5 ml KET buffer (0.5 M KCl; 0.1 M EDTA; and 0.05 M Tris–HCl; pH 8.0) and SET buffer (25 % sucrose, 0.05 M EDTA, and 0.05 M Tris–HCl, pH 8.0) and resuspended in 250 μl of SET buffer containing 5 mg/ml of lysozyme. The mixture was incubated for 10 min at 37 °C. Lysis and purification were completed using the High Pure Plasmid Isolation Kit miniprep (Roche, Switzerland) where the first step was addition of 250 μl of lysis buffer. The original protocol was followed after this step.

### Detection of restriction systems

For identification of putative restriction systems in *C. pasteurianum* NRRL B-598, a protoplast crude extract and whole cell lysate were tested for restriction activity. The whole cell lysate was prepared by sonication (30 min) of the bacterial cells, which were harvested from 30 ml of culture (OD_600_ 0.6–0.8) and resuspended in 5 ml of nuclease-free distilled water. For protoplast preparation, 50 ml of culture (OD_600_ 0.6–0.8) was centrifuged (10,000×*g*, 2 min.), washed with lactose-containing protoplast buffer (25 mM potassium phosphate, 6 mM MgSO_4_, 15 % lactose, pH 7.0) [12, 44] and resuspended in 2–4 ml of protoplast buffer containing 10 mg/ml of lysozyme. The mixture was incubated at 37 °C in the anaerobic chamber for 45–60 min (at least 90 % of cells were transformed to protoplasts). Protoplasts were collected by centrifugation (1200×*g*, 10 min) and lysed in 20 ml TEMK buffer [22] at 37 °C for 1 h after which, cell debris were removed by additional centrifugation (20,000×g, 20 min., 4 °C). The *C. pasteurianum* DSM 525 protoplast crude extract was prepared in the same way as above (15–20 min. cultivation with lysozyme-containing buffer was enough in this case) and used as a positive control in the restriction-system detection assay. Protoplasts and whole cell crude extracts were used immediately for reactions with plasmid DNA.

The reaction mixture composition was the following: 5 μl of protoplast crude extract or whole cell lysate; 0.5 μg of plasmid DNA (pMTL83253 and pMTL82254); reaction buffer added to a final 1× concentration; deionized water was added to a final volume of 20 μl. Reactions were performed at 30 and 37 °C for at least 8 h (4 h in the case of the positive control). After incubation, reactions were analyzed by standard 1 % agarose-gel electrophoresis. Reaction buffers that were tested were the following: commercial R, O, G, B, and Tango buffers for restriction enzymes (Thermo Scientific, USA), a commercial CutSmart buffer for restriction enzymes (NEB, UK) and CpaAI reaction buffer [45].

### Bioinformatics

Bioinformatics analysis was focused on revealing genes for antibiotic resistance, putative restriction barriers and methylation enzymes and motifs in the *C. pasteurianum* NRRL B-598 whole genome sequence.

The methylome was characterized using PacBio Single Molecule Real-Time sequencing (2× SMRT cell) kinetic data collected during the genome sequencing process [46]. SMRT Analysis v.2.3 using “RS_Modification_and_Motif_Analysis.1" protocol was used for genome‐wide base modification and detection of the affected motifs. The default quality value (QV) score of 30 was used for motif determination. The detected motifs were uploaded and further analyzed using the REBASE database [24]. The complete genome was also scanned for homologs of R-M system genes using BLAST searching against REBASE and GenBank databases.

Identification of antibiotic resistance genes was carried out with RGI (Resistance Gene Identifier) version 2 [47]. The predicted ORFs were manually compared to genes in the *C. pasteurianum* NRRL B-598 complete genome [19] predicted by NCBI Prokaryotic Genome Annotation Pipeline (PGAP) (http://www.ncbi.nlm.nih.gov/genome/annotation_prok/) and GenBank accession numbers of protein products of relevant genes were assigned.

Statistics analyses describing transformation efficiency were calculated and visualized using Matlab 2014b.

### Preparation of electrocompetent cells and electroporation conditions

For all electroporation experiments, a GenePulser Xcell™ electroporator including both CE and PC module (BioRad, USA) was used. For preparation of electrocompetent cells, 100 ml of TYA medium was inoculated with different proportions of spores and grown overnight. Following a day’s culture, the competent cells were prepared from cells in late-log to early-stationary growth phase (OD_600_ 1.2–1.4). Bacterial cells were centrifuged (10,000×*g*, 3 min, 4 °C), washed once with an equal volume of chilled electroporation buffer (10 % PEG 8000) and gently resuspended in 1/20 volume of the same buffer. Electrocompetent cells were maintained on ice and used for electroporation immediately.

Into a 0.2-cm gap electroporation cuvette (BioRad, USA), 480 μl of competent cells and 2 μg of plasmid DNA dissolved in 20 μl of demineralized water were mixed and transferred to the electroporator. During optimization of electroporation parameters a Time Constant mode was used. The most successful parameters were the following: 5 ms time constant, 1000 V (corresponding to 50 μF capacitance and 100 Ω resistance). Electroporated cells were incubated for 10 min in the anaerobic chamber on ice and 100 μl of shocked cells were then inoculated into 2 ml of prewarmed and prereduced TYA broth. After 6 h of culture, all cells were harvested by centrifugation, resuspended in 100–500 μl of TYA and plated onto TYA agars with appropriate antibiotic selection, or directly seeded onto plates in different volumes. Growth of antibiotic-resistant colonies was observed after 24–48 h.

All centrifugation and electroporation steps were conducted out of the anaerobic chamber because chamber construction did not allow them to be performed inside.

### Gene transfer by conjugation

*Escherichia coli* HB101 and JM110 both containing helper plasmid RP4 were used as conjugation donors. The donor was transformed by pMTL83253 as described above and conjugation was conducted as described previously [21]. An overnight culture of donor (1 ml) was washed twice with LB broth and 200 μl of overnight recipient culture were added. The mixture was spotted in small drops onto TYA agar medium without antibiotic selection and incubated for 6–24 h. Cells were scraped and washed from the agar with 600 μl of PBS, twice, and plated onto TYA with appropriate antibiotic selection and chloramphenicol or thiamphenicol counter-selection for suppression of *E. coli* donor growth.

### Gene transfer by sonoporation

Sonoporation was performed using a standard laboratory ultrasonic bath (Elmasonic E120H, Elma Schmidbauer GmbH, Switzerland). Competent cells were prepared in the same way as electrocompetent cells (see above) but were finally resuspended in 1/20 volume of sonoporation buffer (30 % PEG 8 000). Into a flat-bottom glass vial, 480 μl of competent cells and 2 μg of plasmid DNA were mixed and immediately sonoporated in the middle of the ultrasound bath for 20 s. Recovery of the mixture was conducted in the same way as during electrotransformation. Growth of antibiotic-resistant colonies was observed after 24–48 h.

### Combined technique for higher transformation efficiency

For the best transformation efficiency, a combination of sonoporation and electroporation was performed. Competent cells and transformation mix were prepared in the same way as during the standard sonoporation procedure; however only 0.25–2 μg of plasmid DNA was used for transformation. Immediately after sonoporation, cells were transferred to the 0.2-cm gap electroporation cuvette and electroporated using a square-wave pulse (5 ms, 1250 V). For recovery of the cells, the standard method was used (see above).

### Statistical and control approaches

All transformation experiments were performed at least three times. Transfer efficiencies of foreign DNA were calculated as an average value derived from three independent experiments. Negative controls (transformation mixture without DNA added or conjugation with donor strain without appropriate a pMTL80000 series plasmid) were used in all transformation experiments.

## Abbreviations

ABE: acetone-butanol-ethanol fermentation
BLAST: basic local alignment search tool
CFU: colony forming units
Dam: DNA adenine methyltransferase
Dcm: DNA cytosine methyltransferase
DNA: deoxyribonucleic acid
m4C: 4-methylcytosine
m5C: 5-methylcytosine
m6A: 6-methylcytosine
MT: methyltransferase
OD_600_: optical density at 600 nm
ORF: open reading frame
PBS: phosphate buffered saline
PEG: polyethylene glycol
R-M system: restriction-modification system
SMRT: single molecule real-time sequencing
